# Microsporidia Infection in a Mexican Kidney Transplant Recipient

**DOI:** 10.1155/2012/928083

**Published:** 2012-12-09

**Authors:** Oscar Xavier Hernández-Rodríguez, Octavio Alvarez-Torres, Norma Ofelia Uribe-Uribe

**Affiliations:** ^1^Department of Pathology, Hospital General Regional No. 6, Instituto Mexicano del Seguro Social, 89210 Madero, TAMPS, Mexico; ^2^Department of Nephrology, Hospital de Beneficencia Española, 89120 Tampico, TAMPS, Mexico; ^3^Department of Pathology, Instituto Nacional de Ciencias Médicas y Nutrición “Salvador Zubirán”, 14000 Mexico City, DF, Mexico

## Abstract

Microorganisms of the microsporidia group are obligated intracellular protozoa that belong to the phylum Microspora; currently they are considered to be related or belong to the fungi reign. It is considered an opportunistic infection in humans, and 14 species belonging to 8 different genera have been described. Immunocompromized patients such as those infected with human immunodeficiency virus (HIV), also HIV serum-negative asymptomatic patients, with poor hygienic conditions, and recipients of bone marrow or solid organ transplantation are susceptible to develop deinfection. Sixty *transplanted* patients with renal microsporidia infection have been reported worldwide. The aim of this paper is to inform about the 2nd case of kidney transplant and microsporidia infection documented in Mexico.

## 1. Introduction

Microsporidia is an intracellular obligated protozoon, which belongs to the Microspora phylum. There are about 140 genera and 1,200 species of microsporidia, which affects human being and animals. It is considered as opportunistic infections in humans, and 14 species from 8 genera have been reported infecting humans. *Encephalitozoon, Enterocytozoon, Pleistophora, Tachipleistophora, Nosema, Vittaforma, Brachiola, and Microsporidium, * the last one includes not classified species [1–3]. 

The identification of these protozoa as an opportunistic pathogen has been described in HIV-infected patients with AIDS, and chronic diarrhea and fever are the most frequent manifestations in Mexican patients with AIDS (present in approximately 30% of the cases) [4]. Most of the intestinal infection is caused by *Encephalitozoon intestinalis* and *Enterocytozoon bieneusi* [2, 5, 6]. Infection by the protozoa has been informed also in healthy people with low socioeconomic status and poor hygienic conditions, mainly who usually drink non-treated water it has been demonstrated waterborne transmission; for this reason, it is considered a potential community risk. This has also been demonstrated in a study in 2 Mexican communities [5, 7].

 Other several cases of microsporidia infection have been described in HIV negative, solid organ transplant recipients. In these cases, it is not clear if the infection was transmitted by the donor or if the immunosuppression favored an infection acquired in the community [8, 9]. There are several diagnostic techniques for microsporidia detection, among them histochemistry and immunohistochemistry; nevertheless, electron microscopy confirms the diagnosis and identifies the species. Complementary studies such as molecular and antigenic probes are very useful [3, 10].

The aim of this paper is to inform an infrequent case of microsporidia nephritis in a kidney transplant recipient.

## 2. Case Report

**Figure 1:**
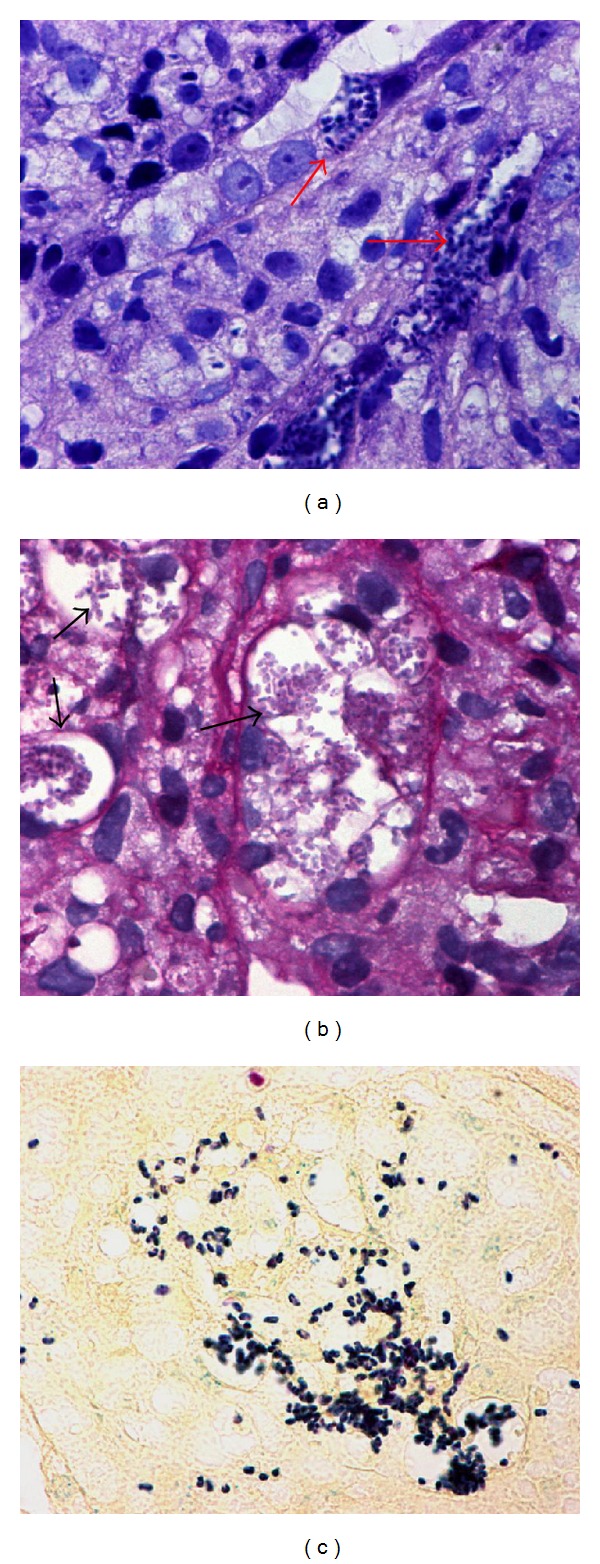
(a) H&E stain: tubular epithelial cells show parasitophorous vacuoles, with abundant microsporidia spores (arrow). (b) Spores are not strongly stained with the PAS stain. (c) Warthin Starry stain strongly stains the spore.

**Figure 2:**
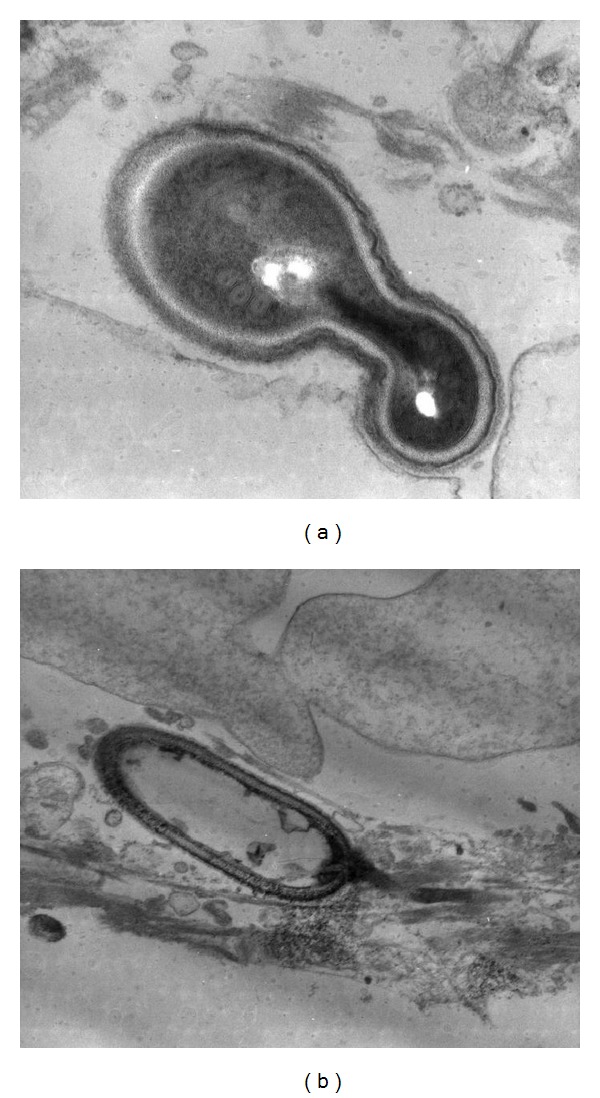
(a) Transmission electron microscopy demonstrates the presence of the meront of microsporidia, with the characteristic coiled polar tube. (b) Microsporidia meront hat shows the polar tube distended.

A 52 year-old male with type 2 diabetes mellitus, who developed end stage renal disease, received a deceased donor kidney transplant. The starting immunosuppressant was Tacrolimus, Prednisone, and mycophenolate mofetil. It is important to mention that the donor had history of drug abuse, it is ignored what kind of drug and time of consumption. The donor died knocked down by a car. The recipient who received the other kidney developed fever and died, and the cause of the fever was not determined. In the present case, the patient developed persistent fever (38.5°C) 5 months after transplantation and renal dysfunction with serum creatinine of 1.2 mg/dL increasing to 4.7 mg/dL at the moment of the biopsy. The urianalysis demonstrated scanty yeast (nonspecific). Acute rejection and possible tuberculosis were suspected. Antibiotics and antituberculosis drugs were empirically given. White blood cell count: 5.14 × 10^3^/*μ*L. Three months later kidney biopsy was taken. Doses of immunosuppressant were tacrolimus 1 mg every 12 hours and Prednisone 10 mg every 24 hours. White blood cell count was 1.8 × 10^3^/*μ*L. The renal biopsy disclosed a granulomatous nephritis without caseous necrosis occupying 60% of the interstitial area, granulomas were observed surrounding tubules, many parasitophorous vacuoles were present in tubular epithelial cells, and within these vacuoles plenty microsporidian spores were identified, but they were also found in the luminal space (Figure 1(a)). The microorganisms were best seen with Warthin-Starry (Figure 1(c)) and modified Gram satins; posterior vacuole was identified with PAS stain (Figure 1(b)). Immunomarking with antibody against *toxoplasma gondii* was negative. Electron microscopy revealed the characteristic ultrastructure of microsporidia spores, exospore, endospore and a coiled polar tubule were identified (Figure 2(a)), and there were also several microsporidia that showed extended polar tube (Figure 2(b)); this finding as well as the development of aninside parasitophorous vacuole is observed in *E. intestinalis, E. hellem, and E. cuniculi* [3]. There were no histological data of rejection.

The patient was treated with albendazole without improvement. Transplant nephrectomy was done, and immunosuppressive therapy was withdrawn; grossly the kidney was edematous, corticomedullary junction was unclear, and a small cortical infarction was identified in the upper pole (possibly related to the previous biopsy). There was an improvement in the patient's condition and fever decrease four days after the nephrectomy. The light microscopy findings were similar to those observed in biopsy.

## 3. Discussion

Sixty cases of microsporidia infection in bone marrow and solid organ transplant recipients have been informed all over the word since 1993 [11–34]. The highest informed prevalence has been reported in France with 40 cases [11–18], 26 of them in kidney transplant recipients, followed by Holland [19] and USA [20–25] with 5 cases each; the following countries have reported 2 cases each: Germany [26, 27], India [28, 29], Spain [30], and Mexico [31] including the present case, both Mexican cases in kidney transplant recipients. One case has been reported in Canada [32], South Africa [33], and Australia [34]. Even when the majority of the cases of microsporidiosis in solid organ transplantation correspond to kidney transplant receptors, kidney involvement is observed in a minority of cases typically as a part of systemic infection [18, 22, 31–33], and only 7 out of 39 informed cases (17%) [22, 30, 33] with kidney involvement manifested with renal dysfunction. 

Clinically, most microsporidia infection presents with diarrhea and weight loss. However, microsporidia have been detected virtually in all organs and may provoke symptoms related to their specific localization, and clinical syndromes associated include enteropathy, keratoconjunctivitis, sinusitis, tracheobronchitis, encephalitis, interstitial nephritis, hepatitis, cholecystitis, osteomyelitis, and myositis [35]. The case from South Africa [33] and the present case had fever and renal dysfunction only, suggesting the affection of this organ only. In the present case, the donor had history of drug abuse, and the recipient who received the other kidney developed fever of unknown origin and died; this antecedent suggests the possibility that the donor had microsporidiosis, and the transplanted kidneys carried out the infection to both recipients. This case, as well as other similar cases, encourages us to consider organ transplant recipients as a risk group for microsporidiosis; a systemic search is recommended for these protozoa in cases of transplant recipients with persistent diarrhea and fever of unknown origin, or any of the above cited syndromes [35]. 

The diagnosis of microsporidiosis is made histologically, either from tissue biopsies or secretions. The development of inside parasitophorous vacuole is observed in *E. intestinalis, E. hellem, and E. cuniculi *infection [3]. Transmission electron microscopy (TEM) was used to confirm the diagnosis, based on revealing characteristic ultrastructural features such as the coiled polar tube within spores and posterior vesicle characteristic of microsporidia [3]. TEM along with special stains and light microscopy, as well as immunohistochemical and molecular techniques, is capable of providing a firm diagnosis, and taxonomic organization of the microsporidia [3, 10]. 

Encephalitozoon species typically are responsible for disseminated infections [3]; *E. intestinalis and E. cuniculi* are the most common microsporidia infecting the kidney [1–3, 8]; from the 7 cases with kidney involvement, 4 (57%) were related with *Encephalitozoon cuniculi*, and one (14%) *E. Intestinalis* [18, 22, 31–33]; in the other cases *Encephalitozoon* was identified, but the species was not determined [34]. Possibly* E. cuniculi *has more avidity for renal tissue [18, 22, 31, 32].

## 4. Conclusions

Not only HIV immunosuppressed patients should be considered at risk for microsporidia infections, but also immunosuppressed patients such as solid organ transplanted patients must be considered in this group also. 

A systemic search is commended for these protozoa in cases of transplant recipients with persistent diarrhea or fever of unknown origin.

This is the second case of microsporidiosis, reported in Mexico, both of them in kidney transplant recipient. The present case had no systemic manifestations of the infection, with transplant dysfunction and fever.
